# Pancreaticobiliary Maljunction Presenting With Choledocholithiasis: A Case Report

**DOI:** 10.7759/cureus.116772

**Published:** 2026-09-23

**Authors:** Oliver G Nicholls, Ioannis Braimakis, James Franklin, Andrew P Pearce, Murad Bayati, Noor Bekkali

**Affiliations:** 1 Translational Gastroenterology and Liver Unit (TGLU), Oxford University Hospitals NHS Foundation Trust, Oxford, GBR; 2 Department of Radiology, Oxford University Hospitals NHS Foundation Trust, Oxford, GBR; 3 Surgical Emergency Unit, Oxford University Hospitals NHS Foundation Trust, Oxford, GBR

**Keywords:** biliary malignancy, endoscopic retrograde cholangiopancreatography (ercp), endoscopic ultrasound (eus), magnetic resonance cholangiopancreatography (mrcp), pancreaticobiliary maljunction (pbm), pancreaticobiliary reflux

## Abstract

Pancreaticobiliary maljunction, an anatomical variant where the common bile duct and main pancreatic duct merge before entering the duodenum, predisposes to pancreaticobiliary reflux with related complications. We present the case of a 69-year-old woman with abnormal liver function tests and progressive hyperbilirubinemia, with biliary dilatation and choledocholithiasis. Magnetic resonance cholangiopancreatography (MRCP) revealed pancreaticobiliary maljunction with choledocholithiasis. Endoscopic ultrasound (EUS) confirmed the presence of two stones within the anomalous common channel, and same-session endoscopic retrograde cholangiopancreatography (ERCP) with sphincterotomy, sphincteroplasty, and balloon extraction achieved complete ductal clearance. Follow-up MRCP demonstrated the resolution of the ductal dilatation. This case highlights the diagnostic value of combined MRCP and EUS in delineating pancreaticobiliary maljunction and stone burden, as well as the role of ERCP in the management of lithiasis. Awareness of this variant is essential to optimize procedural planning, reduce complications, and ensure follow-up, due to the increased biliary malignancy risk associated with pancreaticobiliary maljunction.

## Introduction

Pancreaticobiliary maljunction (PBM) refers to an anomalous union of the common bile duct (CBD) and main pancreatic duct (MPD) outside the duodenal wall. Normally, this is <6 mm in length, but when it exceeds 6 mm, it is considered an anatomical variant, also known as PBM [1]. In PBM, the anomalous junction lies beyond the regulatory influence of the sphincter of Oddi, allowing bidirectional reflux of pancreatic juice and bile between the pancreatic and biliary ducts, with persistent reflux causing epithelial injury and promoting malignancy [1]. PBM is distinct from a choledochal cyst, which is characterized by cystic dilatation of the biliary tree; although PBM is commonly associated with choledochal cysts, it may also occur in the absence of biliary dilatation [1]. Ultimately, PBM leads to pancreaticobiliary reflux (PBR), in which pancreatic secretions reflux into the biliary system and vice versa, due to higher intraductal pressures [2,3].

The clinical consequences of PBM include recurrent pancreatitis, choledocholithiasis, cholangitis, and a documented increase in the risk of biliary tract malignancy, especially gallbladder carcinoma [4,5]. 

The aim of this case report is to highlight the diagnostic and therapeutic challenges associated with PBM presenting with stones within a long common channel. In this setting, we demonstrate the complementary roles of magnetic resonance cholangiopancreatography (MRCP), endoscopic ultrasound (EUS), and endoscopic retrograde cholangiopancreatography (ERCP) in defining the anomalous anatomy, achieving ductal clearance, and informing subsequent multidisciplinary management and malignancy surveillance.

## Case presentation

A 69-year-old woman was admitted under urology for elective left ureteric stent exchange. Her past medical history included endocervical cancer treated with chemoradiotherapy, brachytherapy, and hysterectomy. She had a long-term urinary catheter complicated by right hydronephrosis, ultimately requiring right nephrectomy. A left ureteric stent had subsequently been placed. During this admission, she had abnormal liver function tests (LFTs). These were initially suspected to be secondary to drug-induced liver injury in the context of antibiotic and antifungal use. However, review of prior results demonstrated a progressive cholestatic pattern persisting since 2024.

Abdominal ultrasound revealed intra- and extrahepatic biliary dilatation, with the CBD measuring 12 mm. Several filling defects were identified in the distal CBD consistent with choledocholithiasis. MRCP demonstrated gallbladder adenomyomatosis, biliary and MPD dilatation, and multiple stones within a likely long common channel, raising suspicion of PBM (Figure 1). 

**Figure 1:**
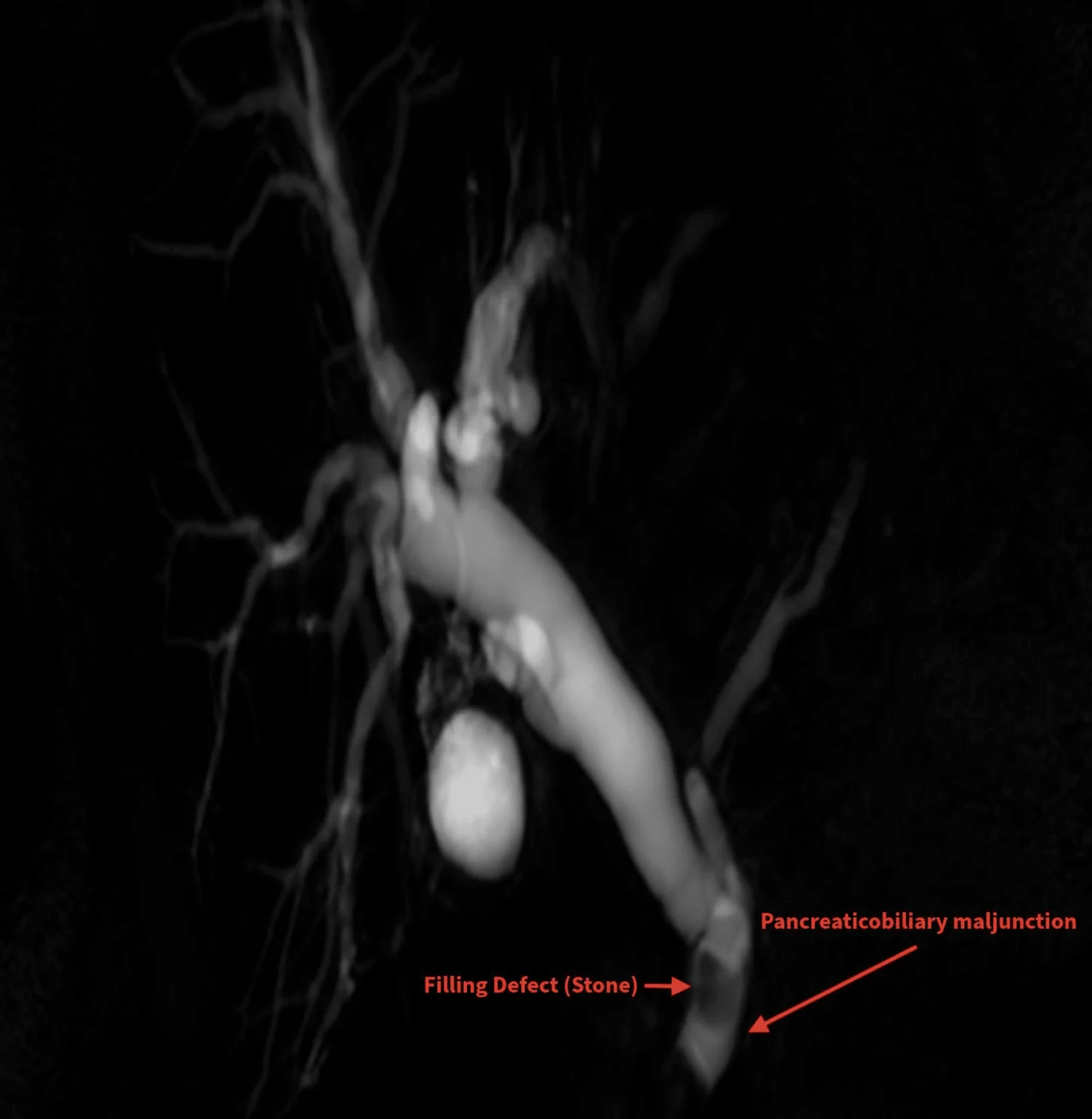
Magnetic resonance cholangiopancreatography demonstrating stones and possible pancreaticobiliary maljunction

Further clarity was sought with EUS which confirmed a dilated CBD (10.7 mm) with two stones within the common channel associated with PBM (Video 1 and Figure 2). Same-session ERCP with sphincterotomy and sphincteroplasty was performed. Trawls with a medium extraction balloon resulted in the complete clearance of the duct (Figures 3-4). 

**Video 1:** Brightness mode endoscopic ultrasound video demonstrating pancreaticobiliary maljunction with two stones within the common channel

**Figure 2:**
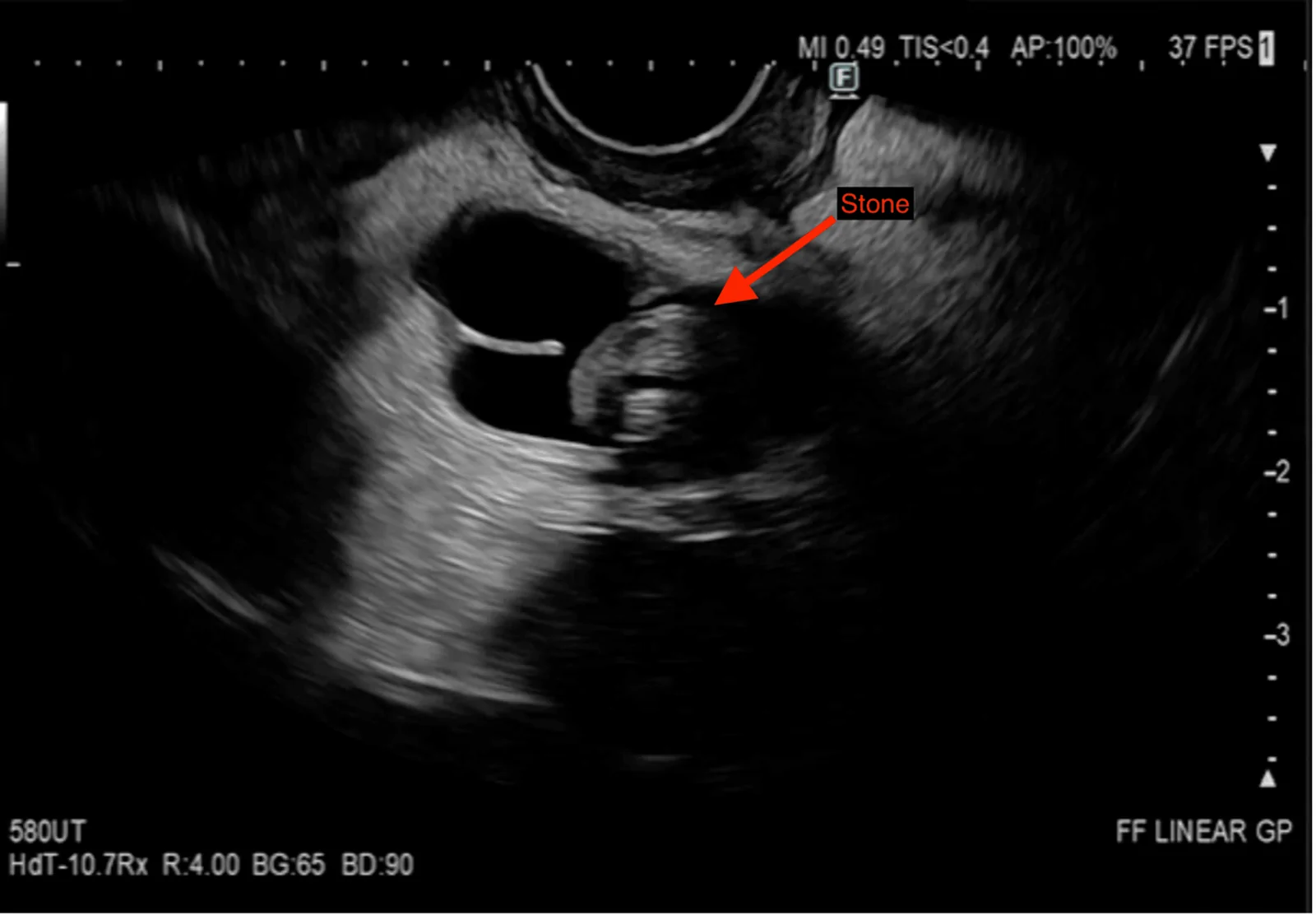
Endoscopic ultrasound (Fujifilm 580UT, Tokyo, Japan) demonstrating two stones within the common channel at the pancreaticobiliary junction

**Figure 3:**
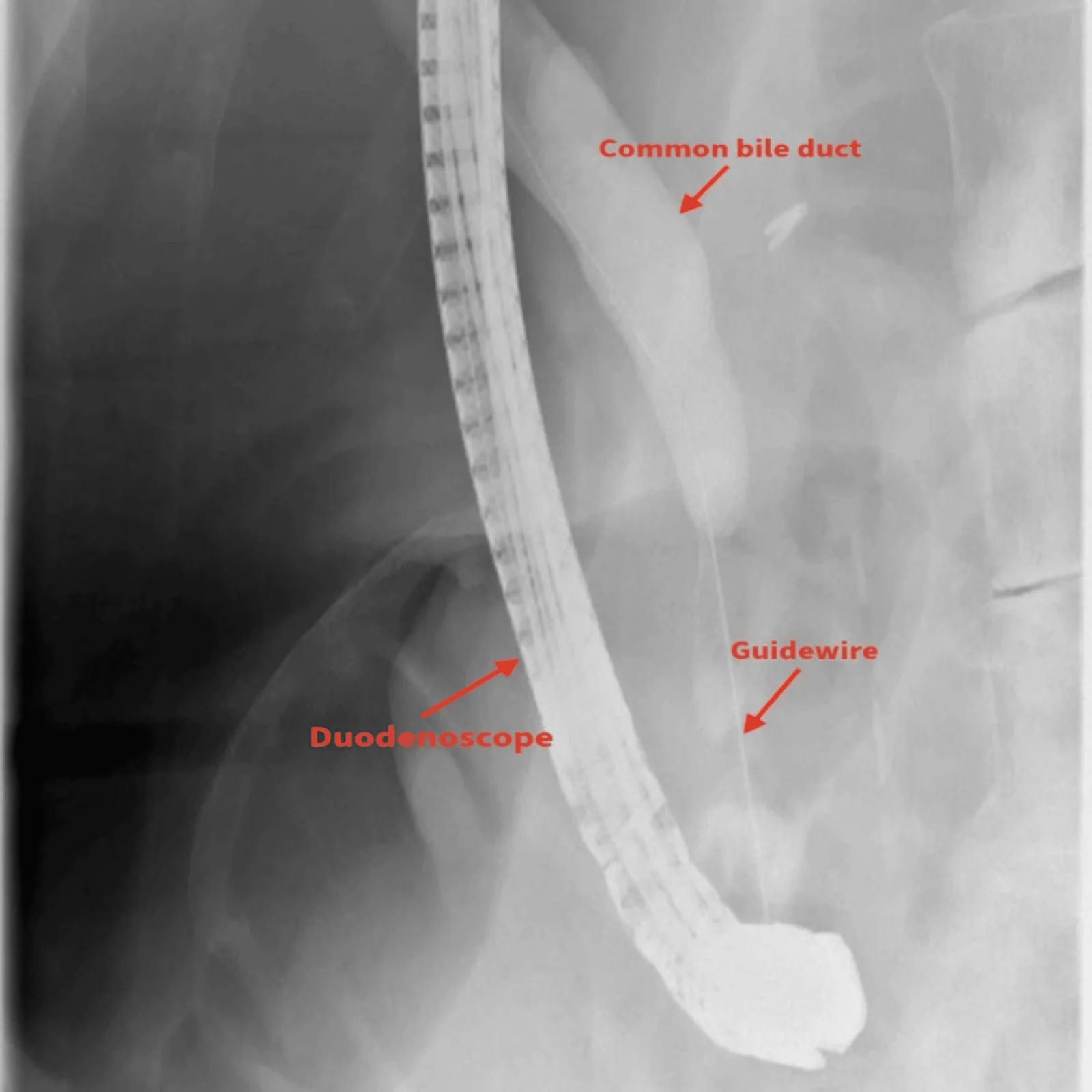
Cholangiogram during endoscopic retrograde cholangiopancreatography demonstrating difficult scope position

**Figure 4:**
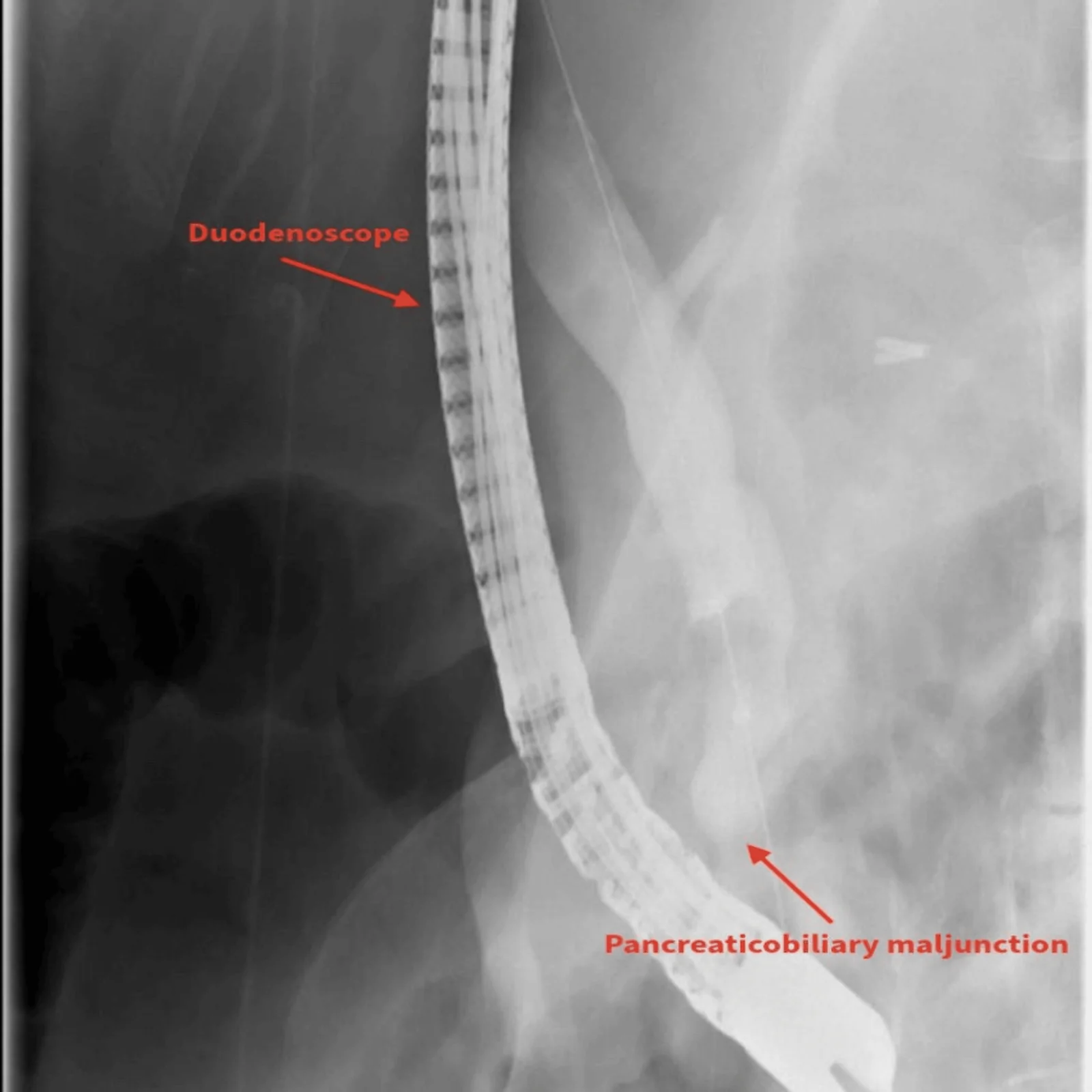
Cholangiogram during endoscopic retrograde cholangiopancreatography demonstrating pancreaticobiliary maljunction and the long common channel

No mass lesion or suspicious stricture was demonstrated on MRCP, EUS, or ERCP. Therefore, brush cytology of the CBD, even though considered, was not performed. The indication for ERCP was the therapeutic clearance of the CBD stones within the long common channel. In our case, there was no clinical, biochemical, or radiological evidence of advanced chronic liver disease. The available liver biochemistry (platelet count (PLT), international normalized ratio (INR), albumin) did not suggest portal hypertension or hepatic decompensation. 

Follow-up MRCP demonstrated the resolution of intrahepatic and pancreatic duct dilatation, with reduction in CBD calibre (Figure 5). Given the increased malignancy risk, the case was discussed with the benign hepatopancreatobiliary team who will review in clinic with repeat magnetic resonance imaging (MRI) and make the consideration of prophylactic cholecystectomy and ongoing surveillance. 

**Figure 5:**
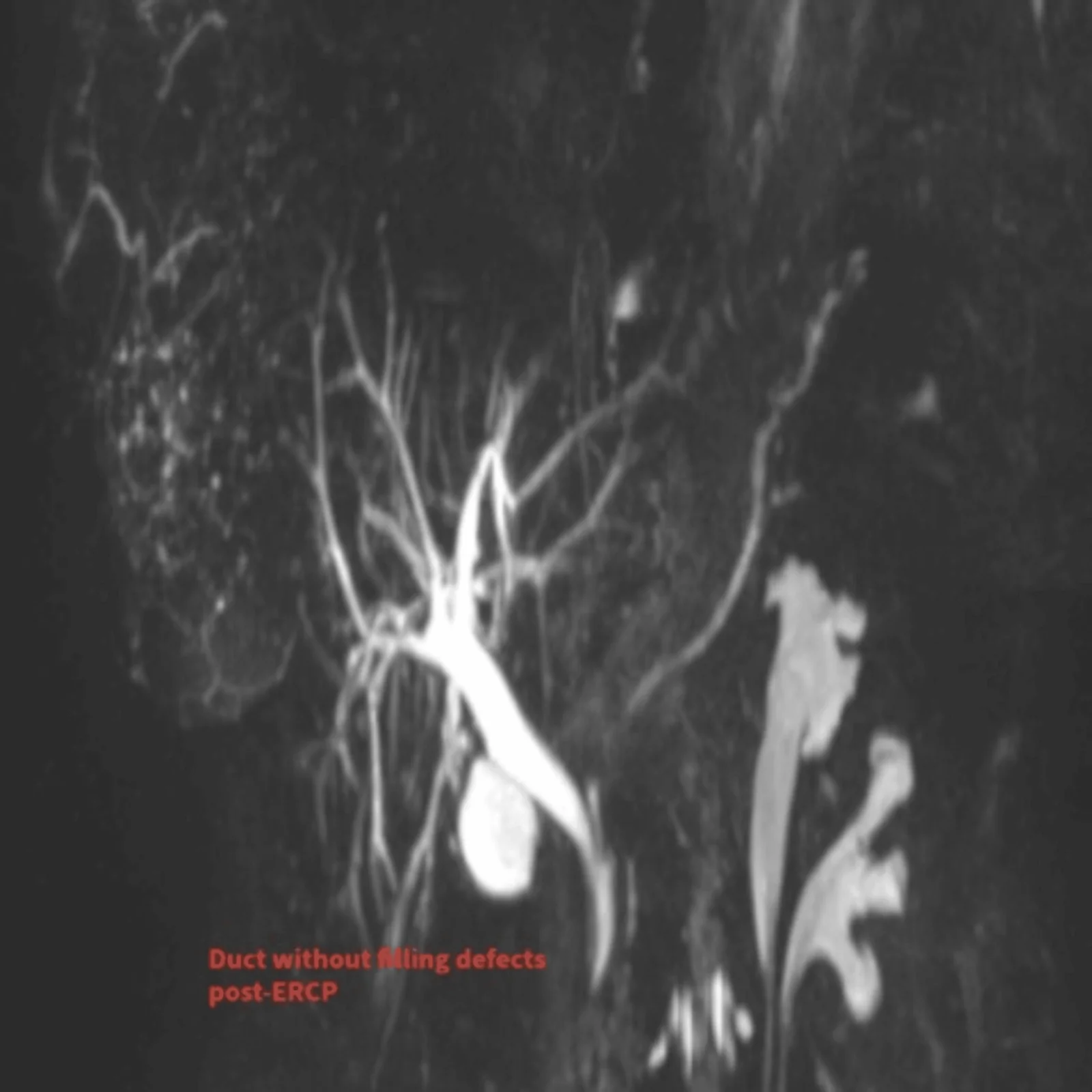
Magnetic resonance cholangiopancreatography post-endoscopic retrograde cholangiopancreatography demonstrating the clearance of ducts from stones

Figure 6 summarizes the imaging modalities and endoscopic interventions in chronological order.

**Figure 6:**
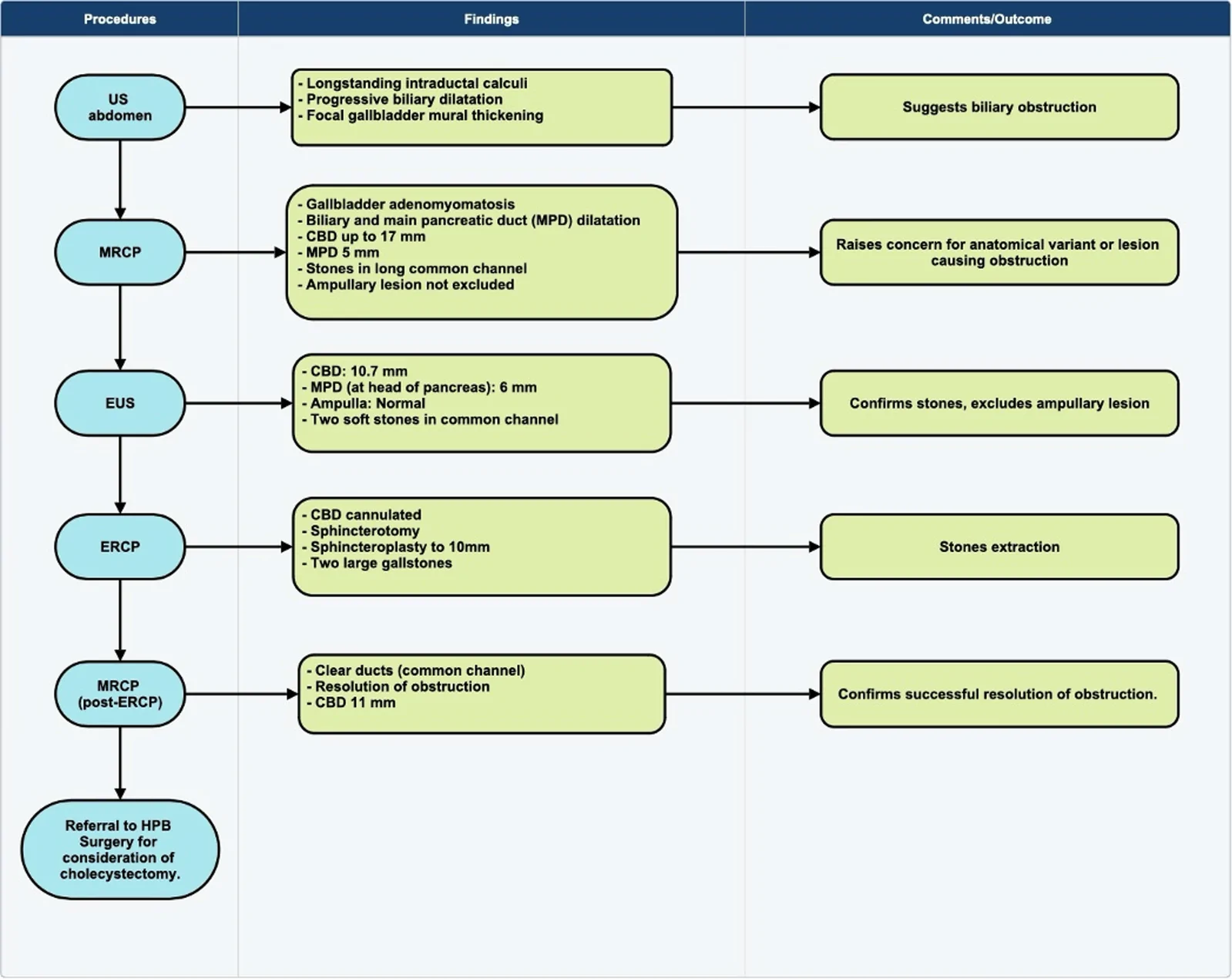
Imaging studies and endoscopic interventions in the work-up of a pancreaticobiliary maljunction presenting with choledocholithiasis EUS: endoscopic ultrasound; ERCP: endoscopic retrograde cholangiopancreatography; MRCP: magnetic resonance cholangiopancreatography; MPD: main pancreatic duct; CBD: common bile duct; HPB: hepatobiliary The figure was created using SmartDraw (SmartDraw, LLC, The Woodlands, Texas, United States).

## Discussion

PBM is commonly identified via MRCP, EUS, or ERCP [1]. The junction typically lies outside the duodenal wall, bypassing the regulatory function of the sphincter of Oddi [1,4]. This allows for PBR, often confirmed by elevated amylase levels in bile samples [3,4].

PBR promotes epithelial injury, predisposing to inflammation, stone formation, and potentially malignant transformation [3,5]. Multiple studies suggest that PBR leads to mucosal changes, hyperplasia, and KRAS mutations, creating a carcinogenic environment, especially in the gallbladder [5]. In addition, stasis and enzymatic imbalance contribute to bile and pancreatic duct stone formation [4]. Moreover, PBMs have been associated with recurrent acalculous pancreatitis [4].

Indeed, recent literature highlights the malignancy risk associated with PBM. A systematic review and meta-analysis including 8604 patients reported that PBM was associated with an 8.4-fold increased risk of any biliary cancer, with the odds ratio for gallbladder cancer (≈16.9) greatly exceeding that for bile duct cancer (≈3.4) [6]. This review further characterized PBM-associated gallbladder cancer, underlining the need for early intervention, i.e., cholecystectomy. A 2025 study showed PBM was present in approximately 16% of gallbladder cancer cases, estimating a gallbladder cancer incidence of 2.4% over 10 years in PBM patients, which was about 38-fold higher than in the general population [7]. In our case, the patient was discussed with the hepatopancreatobiliary team, and repeat MRI with ongoing surgical assessment was planned to determine the appropriateness of prophylactic cholecystectomy and subsequent surveillance; however, it remains unclear how and if we should follow a surveillance pathway similar to other genetic conditions like primary sclerosing cholangitis or hereditary pancreatitis. 

Several expert groups advocate prophylactic cholecystectomy in PBM, especially in fit-for-surgery patients, even without cholelithiasis. In certain cases, additional surgical resection (e.g., of dilated extrahepatic bile ducts) may be warranted. For patients in whom surgery is high-risk or who are discovered incidentally, imaging follow-up for cancer surveillance (e.g., MRCP) might be considered [6,7].

Appropriate diagnostic work-up is critical for decision-making in PBM. MRCP offers excellent imaging of the biliary tree and is highly specific for biliary stones; by contrast, EUS provides direct assessment of the bile and pancreatic ducts [8]. In a recent review and meta-analysis, the pooled sensitivity and specificity for EUS were 96% and 92%, respectively, while MRCP showed 85% sensitivity and 90% specificity for the diagnosis of choledocholithiasis [9]. Furthermore, EUS allows real-time assessment and planning for same-session ERCP if stones are detected [8].

Additionally, EUS allows precise measurement of the PBM, which is critical for management. During ERCP, care must be taken to avoid inadvertent cannulation of the pancreatic duct, since this increases the risk of post-ERCP pancreatitis [10]. Techniques such as wire-guided cannulation, pancreatic duct stenting in cases of inadvertent pancreatic duct cannulation, early pre-cut sphincterotomy, and careful manipulation of the guidewire to avoid pancreatic duct contrast injection can help mitigate this risk [10]. Moreover, stone extraction may require modifications to standard ERCP technique, especially when stones are located proximal to the confluence or within the common channel. 

In our case, both MRCP and EUS identified two stones, but EUS stated the findings unequivocally compared to MRCP. EUS and ERCP enabled the definitive diagnostic characterization of the PBM and successful therapeutic clearance of common channel stones, respectively. Subsequent MRI demonstrated the resolution of the intrahepatic and pancreatic duct dilatation. Following hepatopancreatobiliary multidisciplinary team review, the patient was referred for prophylactic cholecystectomy and ongoing MRI surveillance, reflecting the need for individualized management in an older patient with significant comorbidities. Taking into consideration the patient's comorbidities, surveillance with MRI was proposed as a pragmatic risk-adapted strategy.

## Conclusions

PBM represents an anatomical variant with significant clinical and procedural implications. Its recognition is crucial as it can influence both the diagnostic approach and therapeutic techniques. Accurate delineation of the anatomy, awareness of reflux-related complications, and tailored procedural strategies can help optimize outcomes. Appreciation of this variant also affects post-procedure management, with surgical intervention and long-term follow-up being of paramount importance, due to the high cancer risk. 

In this case, EUS and ERCP enabled the definitive characterization of the PBM and successful clearance of common channel stones, respectively. Subsequent MRI demonstrated the resolution of the intrahepatic and pancreatic duct dilatation. Following multidisciplinary review, the patient was referred for the consideration of prophylactic cholecystectomy and ongoing MRI surveillance, reflecting the need for individualized management in an older patient with significant comorbidities.

## References

[1] Kamisawa T, Kuruma S, Tabata T (2015). Pancreaticobiliary maljunction and biliary cancer. J Gastroenterol.

[2] Kamisawa T, Ando H, Suyama M, Shimada M, Morine Y, Shimada H (2012). Japanese clinical practice guidelines for pancreaticobiliary maljunction. J Gastroenterol.

[3] Beltrán MA (2011). Pancreaticobiliary reflux in patients with a normal pancreaticobiliary junction: pathologic implications. World J Gastroenterol.

[4] Kimura K, Ohto M, Saisho H (1985). Association of gallbladder carcinoma and anomalous pancreaticobiliary ductal union. Gastroenterology.

[5] Misra S, Chaturvedi A, Misra NC (2003). Carcinoma of the gallbladder. Lancet Oncol.

[6] Rivero-Moreno Y, Goyal A, Bolívar V (2025). Pancreaticobiliary maljunction and its relationship with biliary cancer: an updated and comprehensive systematic review and meta-analysis on behalf of TROGSS-the Robotic Global Surgical Society. Cancers (Basel).

[7] Shirai S, Kaneko K, Kato S, Kondo R, Osawa T, Fukami Y, Sano T (2025). Prevalence of pancreaticobiliary maljunction and high confluence of the pancreaticobiliary ducts: true incidence of gallbladder cancer. J Hepatobiliary Pancreat Sci.

[8] Sharma V, Rana SS, Bhasin DK (2015). Endoscopic ultrasound guided interventional procedures. World J Gastrointest Endosc.

[9] Afzalpurkar S, Giri S, Kasturi S, Ingawale S, Sundaram S (2023). Magnetic resonance cholangiopancreatography versus endoscopic ultrasound for diagnosis of choledocholithiasis: an updated systematic review and meta-analysis. Surg Endosc.

[10] Cotton PB, Eisen GM, Aabakken L (2010). A lexicon for endoscopic adverse events: report of an ASGE workshop. Gastrointest Endosc.

